# Evaluation of the effects of lumbosacral dorsal root ganglion pulsed radiofrequency treatment applied via a caudally inserted flexible electrode in radicular pain due to lumbar foraminal stenosis

**DOI:** 10.1371/journal.pone.0336937

**Published:** 2025-12-17

**Authors:** Ender Sir, Alp Eren Çelenlioğlu, Ezgi Can, Gül Didem Batur Sir, Münevver Ece Güven

**Affiliations:** 1 Department of Algology and Pain Medicine, University of Health Sciences, Gülhane Training and Research Hospital, Ankara, Turkey; 2 Department of Industrial Engineering, Gazi University, Ankara, Turkey; Nippon Medical School, JAPAN

## Abstract

**Background:**

This retrospective study aimed to evaluate the effects of unilateral lumbosacral dorsal root ganglion (DRG) pulsed radiofrequency (PRF) treatment administered via a caudally inserted electrode to patients with unilateral lumbar radicular pain (LRP) due to foraminal stenosis.

**Methods:**

The study cohort included 80 patients. Patients were evaluated using the numeric rating scale (NRS) and modified Oswestry disability index (MODI) at pre-, 3 weeks, and 6 months postprocedure. Patients were retrospectively divided into three groups based on their lumbar surgery history: those without a lumbar surgery history (group N), those who underwent discectomy surgery (group D), and those who underwent lumbar stabilization surgery (group S). Treatment success was defined as a ≥ 50% decrease in pain scores at 6 months.

**Results:**

NRS and MODI scores were significantly decreased at all follow-ups (*P* < 0.001). Treatment success (≥50% reduction in NRS scores) at 6-month follow-up was 53.6% in the cohort (group N, 75%; group D, 61.5%; group S, 30.8%; *P* = 0.004). In group N, decreased NRS and MODI scores were more significant than those with a history of surgery, with the procedure duration being shorter.

**Conclusions:**

DRG PRF administered caudally is an effective, safe method in patients with refractory LRP due to foraminal stenosis. Specifically, it provides convenience on patients with instrumentation-related imaging difficulties or those with anatomic disorders such as lordosis, large osteophytes, and severe rotation. In addition, this treatment method can be preferred due to its advantages such as reaching multiple DRG levels with a single needle entry, applying PRF and direct drug injection to the epidural space.

## Introduction

Lumbosacral radicular pain (LRP) is a neuropathic condition with a prevalence of 13–40%, characterized by sensory and motor deficits in the affected dermatome, radiating into the lower back and leg [1,2]. Its most common pathologies are disc herniation, facet hypertrophy, and degenerative changes leading to spinal or foraminal stenosis [3,4]. Conservative approaches such as lifestyle modification, oral medications (nonsteroidal anti-inflammatory drugs, myorelaxants, anticonvulsants), and physical therapy modalities are the first-line treatment for LRP. Interventional pain management options are available for patients with LRP who cannot tolerate oral treatment or dose escalation, or are resistant to conservative treatment [5,6]. Although the standard treatment for radicular pain is epidural steroid injection using interlaminar or transforaminal access [6,7], pulsed radiofrequency (PRF) therapy applied to the dorsal root ganglion (DRG) has provided efficacy similar to the clinical results of steroid injections, and furthermore, has a safer and longer lasting effect on pain relief [8,9]. For PRF treatment, the tissue temperature reaches a maximum of 40°C–42°C, with neuromodulation achieved through an electromagnetic effect rather than a thermal effect in the DRG. This approach prevents potentially irreversible thermal-induced tissue damage and cell destruction [9–11].

In LRP, the classic transforaminal approach is the most common method used to treat DRG with PRF, which is effective for pain management [9]. However, in patients with anatomical abnormalities such as lordosis, large osteophytes, severe rotation, and the presence of metallic screw implants, the classic transforaminal approach is difficult. Consequently, the DRG can be accessed by entering the caudal epidural space through the sacral hiatus using a flexible electrode [12,13]. This approach provides easier and safer access to the epidural space and allows targeting of multiple DRG levels with a single needle entry.

This study aimed to evaluate the effects of lumbar DRG PRF via caudal percutaneus access with a flexible electrode in patients with LRP due to lumbar foraminal stenosis. The primary aim was to determine the effects of treatment on pain by comparing pain scores at pre- and 6 months posttreatment. The secondary aim was to evaluate and compare the effects of treatment on disability and functionality, and the treatment outcomes of patients divided into groups retrospectively according to their surgical status.

## Materials and methods

### Study design and participants

**Fig 1:**
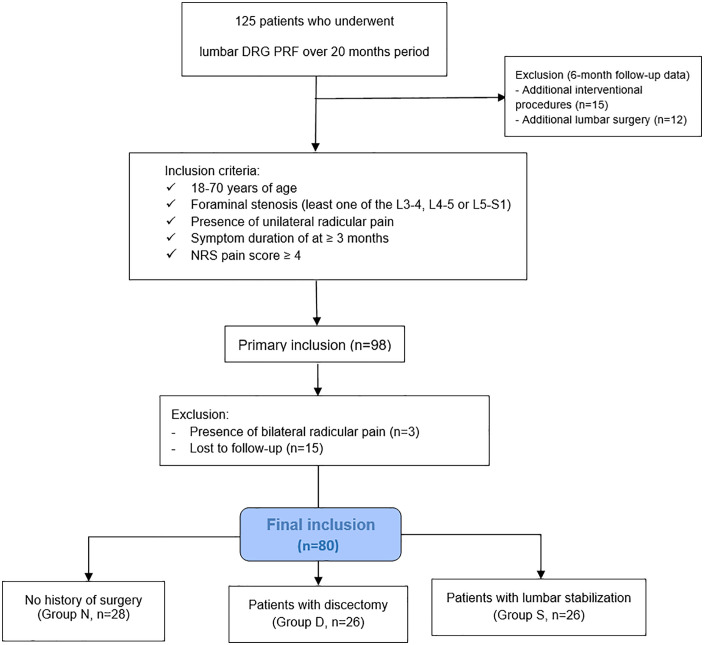
Study design and flow chart diagram.

The data of 125 patients with LRP due to lumbar foraminal stenosis who underwent DRG PRF treatment between January 2022 and August 2023 at a tertiary care center were retrospectively screened. Among them, 80 patients who met the inclusion criteria were included in the study (Fig 1). Patient data were started to be accessed on 01.05.2024 for research purposes and patient data were analysed between 01.05.2024 and 01.06.2024. After data collection, participants had access to information that could identify them. Before the procedure, written consent was obtained from all patients in accordance with the informed consent rules determined by the ethics committee of our hospital. The Institutional Ethics Committee approved our study (No. 2023−132) and was conducted following the Declaration of Helsinki principles.

A detailed physical and neurological examination is performed before the procedure in all patients planned for the procedure, and the procedure levels are decided by evaluating the examination findings together with the lumbar MRI findings (affected nerve root).

The following criteria were used to determine eligibility for inclusion in the study: (1) age between 18 and 70 years; (2) the presence of foraminal stenosis (due to causes such as disc herniation, facet hypertrophy) in at least one of the L3-4, L4-5, or L5-S1 levels, diagnosed by physical examination, clinical findings, and magnetic resonance imaging (MRI); (3) symptom duration of ≥3 months; and (4) numerical rating scale (NRS) pain score of ≥4, (5) presence of mixed-type pain (accompanied by neuropathic features), (6) accessibility of clinical and radiological data.

The following criteria will result in the exclusion from the study: (1) presence of bilateral symptoms, (2) history of interventional procedures in the lumbar region within the last 6 months, (3) more severe axial low back pain rather than radicular component, (4) presence of lumbar spondylolysis–spondylolisthesis, (5) presence of abnormalities in neurological examination (sensory, motor, reflex abnormality), (6) intervention at levels other than L3-4, L4-5, L5-S1, (7) presence of severe spinal stenosis, (8) presence of hepatic/renal failure or coagulopathy, and (9) presence of malignancy.

### Data collection and assessment

Demographic data and clinical characteristics of the patients, including age, sex, body mass index (BMI), symptom duration (in months), history of lumbar spinal surgery (discectomy or lumbar stabilization), number of DRG levels targeted during the procedure, and the duration of the procedure (in minutes) were obtained from the patients’ files. Furthermore, the pain intensity of patients at presentation (preprocedure), 3 weeks, and 6 months postprocedure was evaluated using the NRS (0–10 verbal). A ≥ 50% reduction in pain (NRS) score at 6 months was considered a treatment success. Functional results and disability were assessed using the modified Oswestry disability index (MODI) at baseline and at 3 weeks and 6 months posttreatment. Patients were divided into three groups based on their lumbar surgery history: those without a lumbar surgery history (group N), those who underwent lumbar discectomy surgery (group D), and those who underwent lumbar stabilization surgery (group S).

### Radiological assessment

The degree of nerve compression was assessed using axial T2 and sagittal T1 sequences on MRI. A a modified Pfirrmann grading system was used to grade spinal nerve root compression for central and subarticular disc herniation [14]. Grade 1 denotes that the disc contacts only the nerve root; grade 2, the nerve root is displaced but periradicular cerebrospinal fluid (CSF) or fat is preserved; grade 3, periradicular CSF or fat is obliterated; grade 4, the nerve root is morphologically disrupted. Grades 1 and 2 indicated low-grade nerve root compression, whereas grades 3 and 4 indicated high-grade nerve root compression. When grading the patients’ lumbosacral root compression, the one with the highest grade was accepted. The clinician performing radiological evaluation and those performing the interventional procedures were different. Foraminal stenosis was evaluated by postoperative MRI in the group of patients who underwent lumbar surgery.

### Procedure

**Fig 2:**
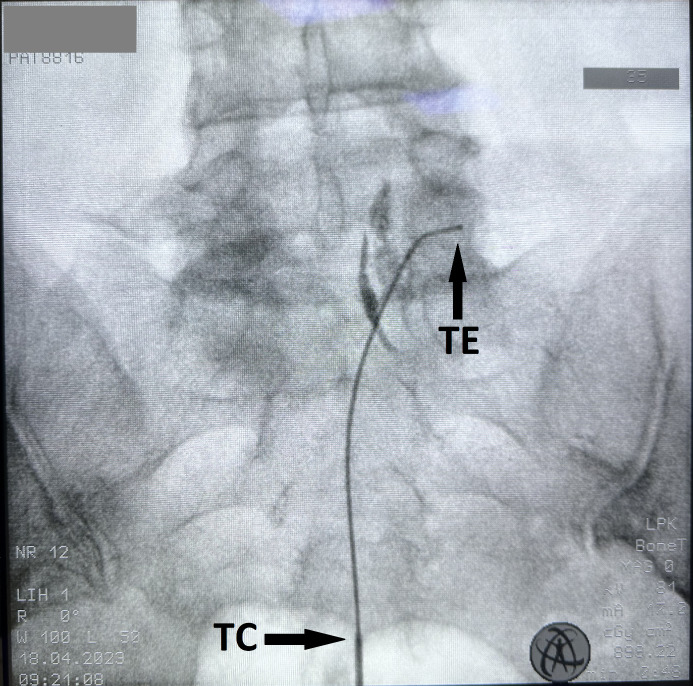
Visualisation of a flexible RCE catheter inserted via caudally at the level of the right L5 dorsal root ganglion, TC: Tip of the cathether TE: Tip of the electrode.

**Fig 3:**
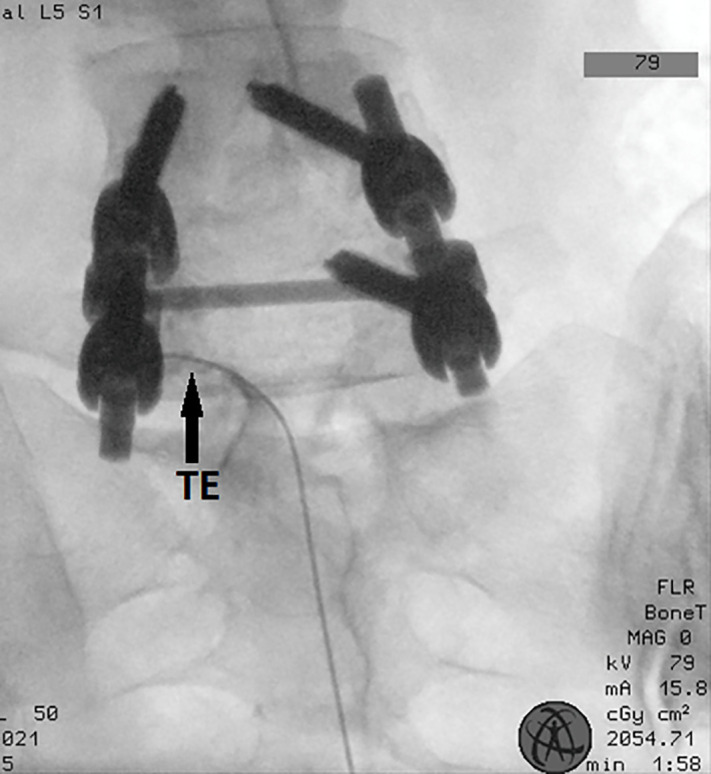
Visualisation of a flexible RCE catheter inserted via caudally at the level of the left L5 dorsal root ganglion, TE: Tip of the electrode.

In the operating room, all patients were placed in the prone position with vascular access and monitored using electrocardiography, oxygen saturation, and noninvasive arterial blood pressure. The skin was cleaned with povidone-iodine. The sacrum was visualized in anteroposterior and lateral view using C-arm fluoroscopy (Ziehm Vision FD). The application of a local anesthetic (lidocaine, 4 cc, 1%) was sufficient to induce skin anesthesia. A 16 G RCE intoducer cannula (Boston Scientific) was inserted through the sacral hiatus. The Tuohy needle was advanced to ensure it did not exceed the S3 sacral foramen. The RCE radiofrequency electrode (40 cm) (Boston Scientific) was advanced to the dorsal epidural space through the Tuohy needle to the intervertebral foramen, where the targeted DRG was located (Fig 2) (Fig 3). The final target for DRG localization was determined as almost mid-level under the pedicle on antero-posterior imaging on fluoroscopy. Once the electrode reached the targeted DRG, sensory stimulation (50 Hz, 0.2–0.5 V) was employed using the Cosman G4 radiofrequency generator (Boston Medical, Burlington, MA) to elicit a paresthesia sensation consistent with the patient’s painful areas. Furthermore, 2 Hz motor stimulation of up to 1 V was performed to elicit muscle contraction in the corresponding myotome area. PRF was applied for 240 s, with the temperature maintained at <42°C. A total of 8 mg dexamethasone, 3 cc lidocaine 2%, and 5 cc saline were injected by dividing equally between the targeted DRG levels. Patients were monitored for 1 h postprocedure to identify any complications. Those who did not experience any complications were discharged.

### Statistical analysis

Descriptive statistics of the measurements were calculated as the means, standard deviations, medians, 25th and 75th quartiles, numbers, and % frequencies. The compatibility of numerical characteristics obtained by measurement with the normal distribution was analyzed using the Shapiro–Wilk test, which found that all variables deviated from the normal distribution. As numerical characteristics were not normally distributed, intergroup and intragroup changes were evaluated using nonparametric tests, such as Kruskal–Wallis test and post-hoc Dunn’s test, to compare the three groups. In addition, periodic changes in NRS and MODI scale scores were evaluated using Friedman’s test, and the period with differences was determined using the post-hoc Dunn test. The relationships between categorical characteristics were analyzed using Pearson’s chi-squared test. Statistical significance was set at P < 0.05, and Statistical Package for Social Sciences (ver. 23) was used for calculations.

## Results

This study was performed using data obtained from 80 patients. Of these, 52 (65%) were females and 28 (35%) were males. At the 6-month follow-up, 56.3% (n = 45) of the patients achieved at least 50% reduction in NRS scores and were characterized as having a successful treatment response (Tables 1 and 2). Their mean age and symptom duration were 58.8 years and 19.4 months, respectively. About 35% (n = 28) of the patients had no history of surgery (Group N), 32.5% (n = 26) had undergone discectomy (Group D), and 32.5% (n = 26) had undergone lumbar stabilization (Group S). In 81.3% (n = 65) of these patients, two DRG PRF levels were performed, whereas 18.7% (n = 15) underwent three or more levels.

**Table 1:** Categorical data on clinical and radiological characteristics in all patients.

		n	%
Etiology	Disc herniation	26	32,5
	Facet hypertrophy	6	7,5
	Both	48	60,0
Grade of nerve root compression	2	22	27,5
	3	58	72,5
Intervention side	Right	39	48,8
	Left	41	51,2
Targeted dorsal roots	2 roots	65	81,3
	≥3 roots	15	18,8
Successful treatment response (6th month)	Yes	45	56,2
	No	35	43,8
Groups according to history of lumbar surgery	No lumbar surgery	28	35,0
	Discectomy	26	32,5
	Lumbar stabilization	26	32,5

**Table 2:** Clinical features of numerical characteristics in all patients and change in scores over time.

	N	Mean	SD	Percentiles		
				25th	Median	75th
Age (years)	80	58,81	7,83	54,00	58,50	65,00
BMI (kg/m2)	80	29,05	4,15	26,27	29,08	31,88
Duration of symptoms (months)	80	19,44	10,09	13,00	16,00	24,00
Procedure time (minutes)	80	33,98	7,65	30,00	35,00	40,00
NRS baseline	80	8,84	0,91	8,00	9,00	9,00
NRS week-3	80	4,38	2,17	2,25	4,00	6,00
NRS month-6	80	4,63	2,51	3,00	4,50	7,00
MODI baseline	80	62,56	8,80	58,00	63,00	68,00
MODI week-3	80	34,60	13,71	24,00	32,00	42,00
MODI month-6	80	34,55	17,20	20,00	34,00	49,50

NRS: Numerical rating scale, MODI: Modified Oswestry Disability Index.

Age, BMI, symptom duration, treatment side, targeted dorsal roots, and baseline NRS and MODI scores were similar among the three groups (*P* > 0.05) (Tables 4 and 5). Both disc herniation and facet hypertrophy were significantly more common in patients with lumbar stabilization (Group S) than in those with discectomy (Group D) and no history of lumbar surgery (Group N) (Table 4; p = 0.025). Similarly, the degree of spinal stenosis was significantly higher in patients who underwent lumbar stabilization (Table 4, p = 0.002).

**Table 4:** Distribution of categorical characteristics of patients according to history of lumbar surgery.

	No surgery		Discectomy		Lumbar stabilization		
	n	%	n	%	n	%	P*
Etiology**							
Disc herniation	15	53,6^a^	8	30,8^ab^	3	11,5^b^	**0.025**
Facet hypertrophy	1	3,6^a^	2	7,7^a^	3	11,5^a^	
Both	12	42,9^a^	16	61,5^ab^	20	76,9^b^	
Degree of foraminal stenosis**							
2	14	50,0^a^	6	23,1^a^	2	7,7^b^	**0.002**
3	14	50,0^a^	20	76,9^a^	24	92,3^b^	
**Intervention side**							
Right	13	46,4	13	50,0	13	50,0	0.955
Left	15	53,6	13	50,0	13	50,0	
**Targeted dorsal roots**							
2 roots	23	82,1	21	80,8	21	80,8	0.989
≥3 roots	5	17,9	5	19,2	5	19,2	
Successful treatment response (6th month)**							
Yes	21	75,0^a^	16	61,5^a^	8	30,8^b^	**0.004**
No	7	25,0	10	38,5	18	69,2	

*:Pearson chi-square test, **: There are completely different letters (such as a and b) next to the % values of the groups that differ significantly.

**Table 5:** Descriptive values of the numerical characteristics of the patients according to the status of lumbar surgery.

		N			Percentiles			P**
			Mean	SD	25th	Median	75th	
Age (years)	No lumbar surgery	28	57,21	5,68	54,00	56,00	60,75	0.250
	Discectomy	26	59,38	9,16	52,50	63,00	67,25	
	Lumbar stabilization	26	59,96	8,38	54,75	64,00	66,25	
BMI (kg/m^2^)	No lumbar surgery	28	29,19	4,57	25,71	29,37	32,33	0.880
	Discectomy	26	28,86	3,71	26,33	29,09	30,54	
	Lumbar stabilization	26	29,09	4,24	27,24	29,07	31,74	
Duration of symptoms (months)	No lumbar surgery	28	18,32	7,57	13,25	16,00	19,00	0.838
	Discectomy	26	19,15	11,46	12,00	16,00	22,50	
	Lumbar stabilization	26	20,92	11,18	12,00	16,50	27,00	
Procedure time (minutes)	No lumbar surgery	28	30,18	6,31	30,00	30,00	30,00	**0.002**
	Discectomy	26	34,92	8,15	28,75	35,00	44,25	
	Lumbar stabilization	26	37,12	6,95	33,75	40,00	40,00	
NRS baseline	No lumbar surgery	28	8,64	,989	8,00	9,00	9,00	0.384
	Discectomy	26	8,96	,824	9,00	9,00	9,25	
	Lumbar stabilization	26	8,92	,891	8,00	9,00	10,00	
NRS week-3	No lumbar surgery	28	3,61^a^	2,061	2,00	3,00	5,00	**0.009**
	Discectomy	26	4,15^a^	1,736	2,75	4,00	5,00	
	Lumbar stabilization	26	5,42^b^	2,335	3,00	6,00	7,00	
NRS month-6	No lumbar surgery	28	3,21^a^	2,217	1,00	3,00	4,75	**<0.001**
	Discectomy	26	4,38^b^	1,941	3,00	5,00	5,25	
	Lumbar stabilization	26	6,38^c^	2,316	4,00	7,00	8,25	
MODI baseline	No lumbar surgery	28	60,04	9,640	55,25	63,00	66,00	0.170
	Discectomy	26	63,38	7,083	58,00	62,00	68,50	
	Lumbar stabilization	26	64,46	9,092	60,00	67,00	72,00	
MODI week-3	No lumbar surgery	28	27,86^a^	11,610	20,00	24,00	32,00	**<0.001**
	Discectomy	26	32,77^a^	9,484	27,00	32,00	40,00	
	Lumbar stabilization	26	43,69^b^	14,759	31,50	41,00	60,00	
MODI month-6	No lumbar surgery	28	25,00^a^	13,283	14,00	22,00	34,00	**<0.001**
	Discectomy	26	31,69^a^	12,518	21,50	32,00	38,00	
	Lumbar stabilization	26	47,69^b^	17,258	34,00	50,00	62,50	

*: Kruskal-Wallis test; **: Different letters next to the averages indicate significantly different groups (such as a and b).

NRS: Numerical rating scale, MODI: Modified Oswestry Disability Index.

**Fig 4:**
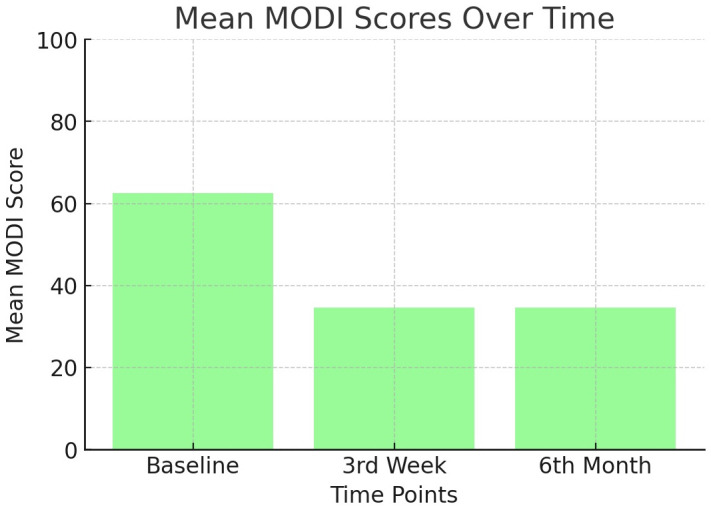
Change in MODI scores over time.

**Fig 5:**
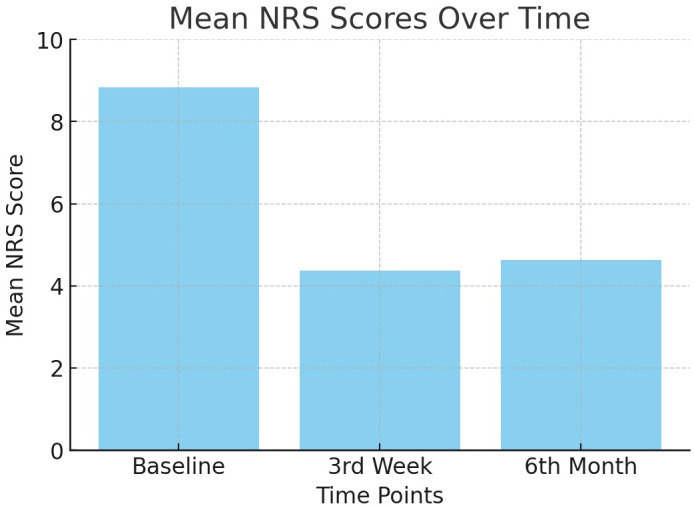
Change in NRS scores over time.

Evaluation of the NRS and MODI scores showed a significant decrease at all follow-up examinations (*P* < 0.001) (Table 3 and Figs 4 and 5). The treatment success rate at 6 months post-treatment was 53.6% in the study cohort (group N, 75%; group D, 61.5%; group S, 30.8%), indicating a significantly lower rate in group S than the other groups (*P* = 0.004) (Table 4). In accordance with this finding, while baseline NRS and MODI scores were similar for the three groups, post-procedural 3rd week and 6th month NRS and MODI scores were significantly higher in patients with lumbar stabilization. Furthermore, the mean procedure time was longer in group S than in the other groups (*P* = 0.002) (Table 5). These findings showed that in patients undergoing surgery (discectomy and especially lumbar stabilization), the efficacy of lumbar DRG PRF treatment via the caudal route was significantly reduced, and the duration of the procedure was significantly prolonged (Table 5).

**Table 3:** Change in NRS and MODI scores over time.

	N	Mean	SD	Percentiles			
				25^th^	Median	75th	P*
**NRS**							
Baseline	80	8,84^a^	0,906	8,00	9,00	9,00	**<0.001**
Week 3	80	4,38^b^	2,172	2,25	4,00	6,00	
Month 6	80	4,63^b^	2,513	3,00	4,50	7,00	
**MODI**							
Baseline	80	62,56^a^	8,801	58,00	63,00	68,00	**<0.001**
Week 3	80	34,60^b^	13,707	24,00	32,00	42,00	
Month 6	80	34,55^b^	17,205	20,00	34,00	49,50	

*: Friedman test and post-Hoc Dunn test; different letters next to the averages indicate significantly different periods (such as a and b). NRS: Numerical rating scale, MODI: Modified Oswestry Disability Index.

In addition, mean baseline MODI scores were 60.04%, 63.38% and 64.46% in groups N, D and S, respectively, indicating disability in all three patient groups. At 6 months after treatment, the mean MODI scores decreased to 25%, 31.69%, and 47.69% in groups S, D, and N, respectively. Patients in Groups N and D experienced moderate disability, while patients in Group S experienced severe disability and had difficulty exercising.

Four patients experienced vasovagal symptoms during the procedure and treated without any complications. No serious complications were observed postprocedures.

## Discussion

This study demonstrated that DRG PRF administered via caudal access with RCE under the guidance of fluoroscopy resulted in a modest and significant reduction in pain and disability scores in patients with LRP due to lumbar foraminal stenosis, and successful pain relief was achieved in 56.3% of the patients and the MODI disability scores decreased from 62.6% at baseline to 34.5% at post-procedural 6 months.

PRF treatment is widely used for the management of chronic pain. It entails the placement of cannulas that generate 500 kHz high-frequency electrical current in proper proximity to neuronal structures [15]. A significant distinction between this method and thermal RF is that the former causes nerve damage, whereas PRF does not exceed the critical nerve tissue-damaging temperature of 42°C[15]. The electromagnetic field generated by the electrode positioned in the proper proximity to the damaged DRG inhibits the conduction of unmyelinated C fibers, thereby suppressing neuronal excitability and action potential development, resulting in an analgesic effect [16]. The electromagnetic field produced by PRF stimulation induces microstructural alterations of the neural tissues, resulting in long-term synaptic depression and the prevention of harmful signal transmission to the brain [17]. Furthermore, several studies have reported that PRF reduces microglial activation in the dorsal horn and enhances inhibitory pathways by increasing c-fos and transcription factor 3/ATF3 levels in laminae 1 and 2 while decreasing calcitonin gene-related peptide/CGRP levels. This finding represents another potential mechanism [12,18–20]. Additionally, it has been demonstrated that the levels of proinflammatory cytokines measured locally after PRF stimulation are reduced. The neuromodulatory effect caused by the reduction in cytokine levels, such as IL-1 and TNF-α, is thought to contribute to the clinical effect of PRF [21,22]. As in this study, the impact on pain relief and patient functionality during the 6-month follow-up period may be explained by the immunomodulatory and anti-inflammatory properties of PRF [21–23].

The cornerstone of interventional treatment of radicular low back pain due to disc herniation is epidural steroid injection, which can be administered caudally, transforaminally, and interlaminarly [8]. Although epidural steroid injections for LRP appear to be effective in the short term, evidence regarding long-term pain relief remains unclear [24]. Recently, lumbar DRG PRF treatment, commonly administered for radicular low back pain, may provide longer-term effects than transforaminal epidural steroid (TFES), potentially owing to immune modulation in the spinal nerve and DRG [18–20]. Similarly, patients who received PRF following TFES reported prolonged pain relief compared with those who received TFES alone [25]. The classic transforaminal approach has been reported as the most common approach for the effective treatment of DRG with PRF [9]. A previous study reported that the success rate of PRF treatment for LRP using the transforaminal approach varies from 30% to 60% [25]. These studies evaluated the effects of DRG PRF using a transforaminal approach with a rigid needle. In our study, targeted DRGs were stimulated using a flexible epidural RF electrode via percutaneous caudal access to the epidural space. A successful pain relief was achieved in 56.3% of the patients at the 6-month follow-up. Thus, direct access to the epidural space via the caudal route and the use of flexible electrodes provide closer stimulation of the DRG, higher electric field intensity around the DRG, and direct drug infusion into the epidural space than the transforaminal route. Complications of caudal access include infection, epidural hemorrhage, hematoma, dural puncture, and toxic reactions associated with bone damage. Therefore, caudal access is not recommended in patients with infection, tumors at the injection site, bleeding, or coagulation disorders [5].

The number of previous studies reporting the effects of epidural PRF for the treatment of lumbar radicular pain is limited. Vigneri et al. evaluated the effect of DRG PRF via caudal access in 34 patients with chronic lumbosacral radicular pain with neuropathic features. Their results revealed that half of the patients achieved >30% pain relief at the 6-month follow-up [12]. Voloshin conducted a case series of seven patients with 1-year results of DRG PRF via caudal access in patients with persistent radicular pain after epidural steroid injection and transforaminal DRG PRF [13]. They reported that the duration of pain relief lasted from 4 months to 1 year in four patients. Thus, the treatment represents a long-term improvement in patients with intractable radicular pain [13]. Another report indicated that DRG PRF via caudal access can be used as a patient selection step for chronic neuromodulation in patients with LRP, with a success rate of 63.8% and a complication rate of <1% [28]. These studies evaluated the success of DRG PRF treatment via caudal access in the general population of patients with radicular lumbar pain. Our study was superior because we evaluated the treatment success rates according to the surgical status of the patients and the demonstration that the presence of lumbar stabilization has a significant negative effect on the treatment success.

Another factor that may have an impact on the long-term effect of PRF treatment is the number of lumbar DRG levels to which PRF is applied. A retrospective study analyzing 61 patients who underwent PRF at a single lumbar level found a success rate of 29% at 2 months posttreatment, which decreased to 13% at 12 months [26]. The low success rates observed in this study were attributed to the use of a single-level PRF. Therefore, a two-level PRF approach was recommended to reduce the potential for inflammation in adjacent DRGs[26]. With increased number of DRG levels to which PRF is applied, the number of interventions also increases because of the need to re-needle each level, which decreases patient comfort and prolongs procedure time. The use of the DRG PRF via caudal access in this study, which was applied by entering the epidural space through the caudal route using flexible electrode, allows treatment at multiple levels with one needle entry. This increases patient comfort, reduces the procedure time, and makes it safer.

The effect of a history of lumbar surgery on the success of DRG-PRF treatment has been evaluated, and studies have yielded inconsistent results. Some previous studies have found no significant difference in NRS scores after transforaminal PRF between patients with and without surgery for LRP [25,27,28]. Conversely, Abejon et al. reported that PRF was more effective in previously nonsurgically treated patients with LRP due to disc herniation [29]. In our study, patients who underwent lumbar surgery (lumbar discectomy or stabilization) had significantly lower treatment success rates and higher disability scores than those who did not. This may be due to the possible advanced stages of the disease and structural and physiological changes caused by surgery. In addition, in patients with a history of lumbar discectomy or stabilization, dural adhesions may occur because of surgery and may have difficulty accessing the catheter to the targeted dorsal root ganglion. This is also a potential condition that may interfere with the efficacy of PRF treatment, but in this study, the targeted roots were reached in all patients included in the study. Furthermore, the treatment success rate may be reduced by the higher degree of spinal nerve root compression due to foraminal stenosis in group S. In contrast to transforaminal PRF, our study firstly assessed the effects of surgical history on the outcomes of patients undergoing DRG PRF via caudal access. Although surgical history negatively affected the treatment success of DRG PRF in our study, we believe that this method is a simple and advantageous in patients with metal screw implants, where transforaminal access to the nerve root is difficult or impossible.

The main limitation of the study was its retrospective and non-controlled design. Another limitation was that the follow-up period was only 6 months, and long-term evaluation could not be performed. In addition, clinical data on the improvement in the analgesic use could not be obtained from patient records. Another limitation of this study is the application of a mixture of local anesthetics and corticosteroids to the dorsal root ganglion of all patients. Consequently, the treatment results reflect a combination of the analgesic and anti-inflammatory effects of the drugs administered with PRF, rather than the efficacy of PRF treatment alone.

This study demonstrates that fluoroscopy-guided lumbar DRG PRF via caudal access is an effective and safe method for managing pain and reducing disability during LRP treatment for foraminal stenosis, with no serious complications observed post-procedure. Furthermore, these beneficial results were maintained for 6 months in 53.6% of patients. The treatment effect in patients with a history of lumbar surgery is considerably reduced, and the procedure duration is extended in patients with DRG that cannot be reached by the transforaminal approach (such as metallic screws and anatomical abnormalities). DRG PRF treatment with a percutaneous caudally inserted flexible electrode may be a preferable option for LRP management. It provides access to DRGs at multiple levels with a single-needle entry, and direct drug injection to the epidural space and PRF application to the targeted DRG is possible. In addition to the current findings, prospective evaluation in different patient groups, with variable PRF voltage ranges, larger sample sizes and longer follow-up periods are required to evaluate long-term outcomes. Studies evaluating the long-term treatment outcomes of patients who received PRF to the DRG alone would also be valuable in understanding the isolated effect of PRF more clearly. Therefore, no data exist on the longer-term effects of PRFs, which could be beneficial to investigate in future studies.

## Supporting information

S1 Data Data set. (XLSX)
